# From Eutrophic to Mesotrophic: Modelling Watershed Management Scenarios to Change the Trophic Status of a Reservoir

**DOI:** 10.3390/ijerph110303015

**Published:** 2014-03-12

**Authors:** Marcos Mateus, Carina Almeida, David Brito, Ramiro Neves

**Affiliations:** MARETEC, Instituto Superior Técnico, Universidade Técnica de Lisboa, Av. Rovisco Pais, 1049-001, Lisboa, Portugal; E-Mails: carina.almeida@ist.utl.pt (C.A.); david.maretec@ist.utl.pt (D.B.); ramiro.neves@ist.utl.pt (R.N.)

**Keywords:** watershed modelling and management, pollution load reduction, eutrophication, water environmental capacity, land use, water resources management, CE-QUAL-W2 model, SWAT model

## Abstract

Management decisions related with water quality in lakes and reservoirs require a combined land-water processes study approach. This study reports on an integrated watershed-reservoir modeling methodology: the Soil and Water Assessment Tool (SWAT) model to estimate the nutrient input loads from the watershed, used afterwards as boundary conditions to the reservoir model, CE-QUAL-W2. The integrated modeling system was applied to the Torrão reservoir and drainage basin. The objective of the study was to quantify the total maximum input load that allows the reservoir to be classified as mesotrophic. Torrão reservoir is located in the Tâmega River, one of the most important tributaries of the Douro River in Portugal. The watershed is characterized by a variety of land uses and urban areas, accounting for a total Waste Water Treatment Plants (WWTP) discharge of ~100,000 p.e. According to the criteria defined by the National Water Institute (based on the WWTP Directive), the Torrão reservoir is classified as eutrophic. Model estimates show that a 10% reduction in nutrient loads will suffice to change the state to mesotrophic, and should target primarily WWTP effluents, but also act on diffuse sources. The method applied in this study should provide a basis for water environmental management decision-making.

## 1. Introduction

Water quality in rivers and reservoirs is controlled by numerous anthropogenic and natural factors [1,2]. Significant efforts have been made over the last decades to develop water management strategies to ensure a good water quality [3]. Watershed management plans, based on land use restrictions and controlling point source pollution, seek to reduce or eliminate sources of potential risk before they are introduced into the watershed. Together, the land use and land cover, human activities (pressures) and natural factors compromise many ecosystem services in a watershed [4,5]. As such, it is essential to understand the effects of land use activities on water resources to achieve proper management decisions and, consequently, environmental improvement [6].

**Figure 1:**
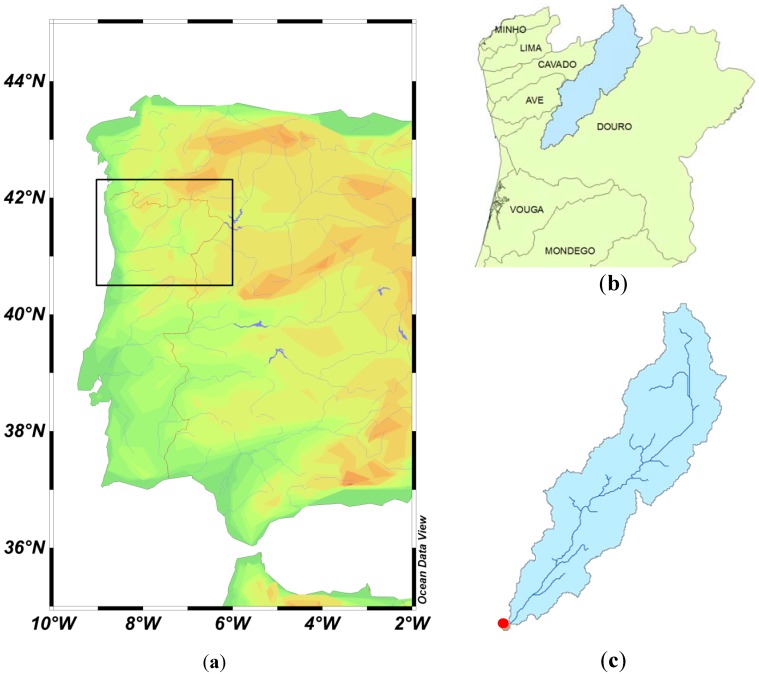
The study area: The black box in the map of Iberia (**a**) outlines the north of Portugal (**b**) where the Torrão drainage basin is located (in blue). (**c**) Caption of the drainage network in the watershed, and location of the reservoir (red dot).

Management decisions related with the water quality in lakes and reservoirs require a combined land-water processes study approach. The complexity of these systems and their interrelated compartments can only be adequately addressed with numerical models [7,8,9]. This study provides such an example for the Torrão reservoir and watershed in Portugal (Figure 1).

The European Water Framework Directive sets actions with the objective of reaching a good ecological status of surface waters until 2015. Water quality models have been shown to be effective tools to address complex water quality problems, and play an important role to provide quantitative information to make best possible decisions in water resources management [9,10,11,12,13].

Nutrient enrichment in reservoirs poses a risk to human health by creating the conditions for the proliferation of nuisance and toxic algae, such as harmful cyanobacterial blooms [2,3]. Such occurrences have been reported in Portuguese reservoirs. For water quality management purposes it is intended to quantify the reduction in the nutrient loads needed to change the actual eutrophic state of the reservoir to mesotrophic. Furthermore, the weight of WWTP on the trophic level is assessed to determine where to act in the basin to reduce the nutrient loads reaching the reservoir (WWTP, agricultural or both). These objectives were accomplished using a combined watershed-reservoir modelling approach. 

This paper is structured in five parts. After the introduction, the study area and model details are explained in Section 2. In Section 3, the modelling methodology will be explained. Section 4 will deal with the model performance and the discussion of the results. Finally, the paper ends with some conclusions in Section 5.

## 2. Material and Methods

### 2.1. Study Area

**Figure 2:**
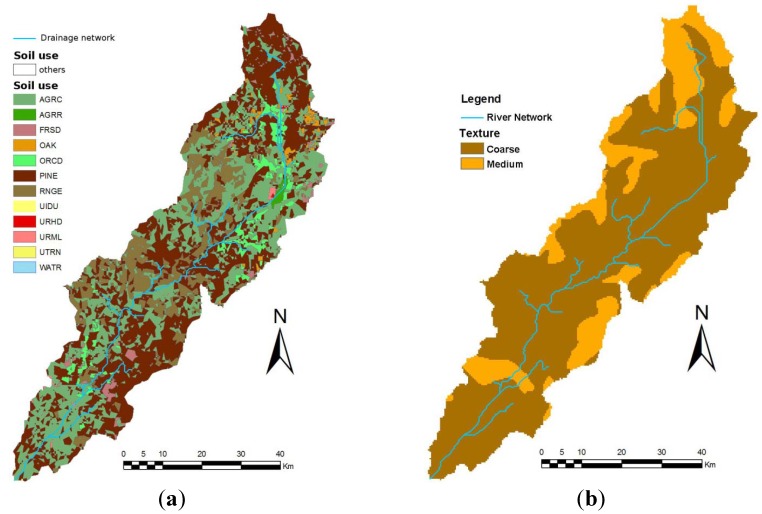
(**a**) Land use in the Torrão watershed according to the CORINE 2000 map. (**b**) Soil texture in the Torrão watershed according to the European Soil Bureau.

The Torrão reservoir is located in the Tâmega River, a tributary of the Douro River. The reservoir has a total capacity of 77 hm^3^ at its maximum filling elevation (65 m), and a mean depth of 19 m. According to the Corine 2000 land cover map, the Torrão reservoir watershed, with approximately 3,280 km^2^, is characterized by a variety of land uses, where pine forest is predominant (accounting for 43% of land occupation), followed by agricultural uses with around 38% of the total area (33% by cold season annual crops and 5% by orchards), and range brush with about 14% of the area (Figure 2a). Human occupation (urban and industrial) accounts for less than 1% of the total area. Soil texture is mostly coarse (79% of the basin, ~2,585 km^2^) and medium grain size (21%, ~695 km^2^) as shown in Figure 2b.

Socioeconomic development in the watershed has led to increased emissions of wastewater and pollutants from domestic, industrial and agriculture sources, affecting the water quality in the Tâmega River and its tributaries. Several urban areas spread over the watershed, with some major urban poles along the Tâmega River. The Torrão basin includes the municipalities of Amarante, Boticas, Cabeceiras de Basto, Celorico de Basto, Mondim de Basto, Chaves, Marco de Canaveses, Montalegre, Penafiel, Ribeira da Pena, and Vila Pouca de Aguiar. Most of these municipalities have several WWTP discharging into the Tâmega river drainage network, accounting for approximately 100,000 p.e.

Torrão reservoir was classified as eutrophic, according to the National Water Institute (INAG), based on the criteria adapted from [14] during the WWTP directive transposition [15]; the geometric mean of surface chlorophyll-*a* field data (from 1996 to 2009) from April to September was 10.6 μg/L, above the threshold of 10 μg/L [15]. Surface field data for Torrão reservoir shows significant inter-annual variation, mostly due to the different yearly rain regimes. This difference relies mostly in the range of values, since there is a clear seasonal pattern repeated in most of the years, consisting of a bigger phytoplankton bloom in spring, followed by a smaller one in summer. Chlorophyll concentration values usually peak between 20 and 30 μg/L chlorophyll-*a* in full bloom, but higher concentrations are not infrequent (see Table 1). 

A summary of the most relevant water quality parameters in the reservoir is presented in Table 1. Higher values of nitrate (1 mgN/L) usually occur during autumn and winter, associated with the increase of water flow in the watershed. Nitrate values are usually lower (<0.5 mgN/L) in spring and summer, usually associated with phytoplankton uptake. Ammonia and phosphate shown no obvious seasonal variation, and values are usually bellow 0.3 mgN/L and 0.05 mgP/L, respectively. The concentrations of both nutrients seem to be related with internal processes in the reservoir, and not so much with the river loads. 

**Table 1:** Description of more relevant water quality parameters at the surface in the Torrão reservoir. Boxplots provide additional information on the frequency of variation for each parameter. Data from 1996 to 2009. 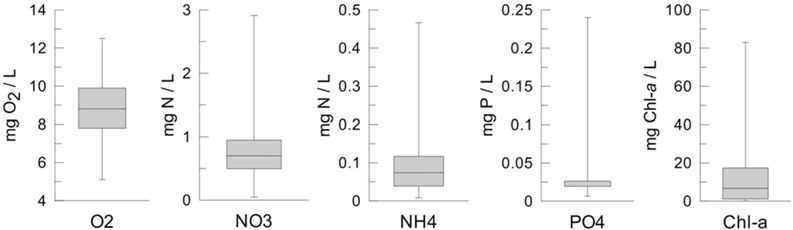

	Dissolved Oxygen (mg/L)	Nitrate (mgN/L)	Ammonium (mgN/L)	Orthophosphate (mgP/L)	Chl-a (ug/L)
**Mean**	8.7	0.77	0.09	0.03	11.5
**Minimum**	5.1	0.05	0.01	0.01	0.0
**Maximum**	12.5	2.91	0.47	0.24	82.9

### 2.2. Field Data

Data used in this study were collected from publically available data sets provided by the Water National Institute (Instituto Nacional da Água, INAG) and specifically from the National Service for Information on Hydric Resources (Serviço Nacional de Informação de Recursos Hídricos, SNIRH, http://snirh.pt). 

### 2.3. CE-QUAL-W2 (CQW2) Model

The CQW2 model was applied to Torrão reservoir to: (i) reproduce the reference situation and, subsequently, (ii) simulate load reduction scenarios to quantify the total maximum input load and possible management scenarios. Total maximum input load is the input load that allows the reservoir to be classified as mesotrophic, instead of eutrophic (reference situation). 

The CQW2 model has been widely used in the study of stratified water systems, including lakes, reservoirs and estuarine environments [7,16,17,18,19,20,21,22]. CQW2 is a two-dimensional (longitudinal-vertical) hydrodynamic and water quality model. The model was originally developed by the U.S. Army Corps of Engineers [23], and a comprehensive description of CQW2 can be found in [24]. CQW2 is particularly adequate to simulate long and narrow water bodies since it assumes lateral homogeneity, implying that there are no significant lateral variations of water quality constituents.

The model uses a direct coupling between hydrodynamic and water quality simulations, assuming the same time step and spatial grid for both sets of processes. The model is based on a finite-difference approximation to the laterally averaged equations of fluid motion, and quantifies free surface elevation, pressure, density, vertical and horizontal velocities, and constituent concentration and transport. Explicit numerical schemes are employed to compute velocities, controlling the transport of energy and biochemical constituents. CQW2 water quality module simulates a number of constituents ranging from a conservative tracer, suspended solids, total dissolved solids, labile and refractory dissolved organic matter, detritus, total phosphorous, orthophosphate, ammonia, nitrate, pH, sediment, oxygen, biochemical oxygen demand (BOD) and several groups of algae. In addition, it is possible to select a subset of interrelated constituents in each simulation.

### 2.4. SWAT Model

SWAT is an integrated model for simulating different watershed processes [25]¸ extensively used over the past years [26,27,28,29,30]. It is a basin-scale, distributed and continuous-time model, and its land hydrodynamic component solves water balance and relates the meteorological variables with basin features such as topography, soil type and land use/vegetation data. Also, the model has a water quality component, accounting for plant growth, nitrogen and phosphorus soil cycles, and sediment and pesticides transport. The SWAT model divides the watershed into sub-basins and into hydrological response units (HRU) that have homogeneous soil, land use and slope characteristics. These are the basic computation units for the simulations. 

The SWAT model solves the water balance between the infiltration/runoff generation (e.g., the modified SCS curve number method), percolation (if the water content is higher than the field capacity), lateral flow (dependent on slope), evapotranspiration and aquifer recharge. The reference evapotranspiration method used in this study was the Penman-Monteith method [31]. The nutrient component of the SWAT model includes inputs from agriculture, transport with runoff and groundwater, consumption by plants and generation by mineralization in the soil.

## 3. Application of the Integrated Model System to Simulate the Tâmega River

### 3.1. Input Data

Data used in the modelling approach can be divided into two categories: implementation data (for running the model), and validation data (to evaluate model performance). 

Atmospheric data was retrieved from the National Service for Information on Hydric Resources (Serviço Nacional de Informação de Recursos Hídricos, SNIRH, http://snirh.pt), for stations located in the Entre-os-Rios, Marco de Canavezes and Penafiel municipalities.

**Figure 3:**
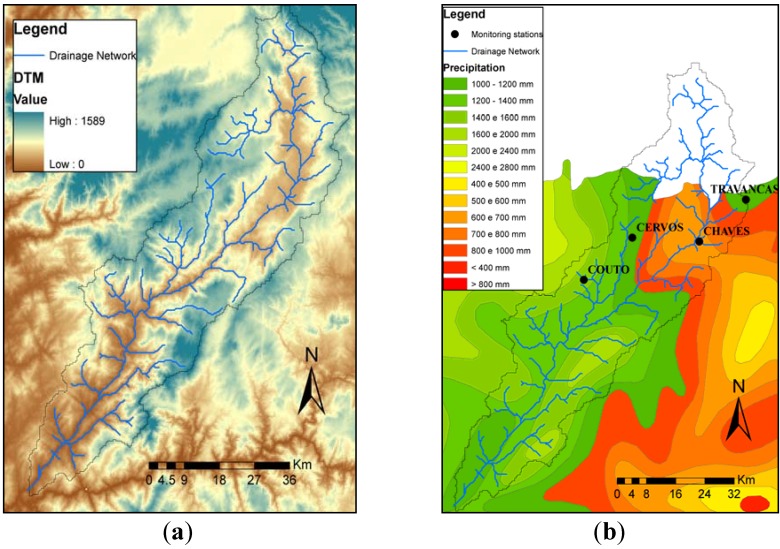
(**a**) Digital Terrain Model (DTM) for the Torrão watershed (Source: NASA). (**b**) Rainfall map for the watershed; dots mark the location of the monitoring stations (Source: INAG).

The watershed was characterized using the Digital Terrain Model from NASA with 90 m spatial resolution [32], as seen in Figure 3a. Precipitation data were collected from the SNIRH monitoring system, for station located within the watershed. The precipitation map is presented in Figure 3b and the mean annual values for the time series used in the study are summarized in Table 2. 

Available data for flow and water quality in the watershed either lack temporal resolution, continuous time series, or both, to allow a proper determination of mean annual nutrient loads reaching the reservoir. As such, river flow and nutrients and organic matter loads were calculated using the SWAT model. WWTP contributions were considered as point sources of urban pollution in the model, and were quantified for each municipality in terms of person equivalent, assuming a release of 100 L/(p.e.·day) of wastewater with a nutrient concentration calculated according to the Portuguese legislation (Table 3). Two WWTP account for more than 10,000 inhabitants (in Amarante and Chaves municipalities) but tertiary treatment is absent. 

**Table 2:** Mean annual precipitation for values measured in the monitoring stations depicted in Figure 3. Data for the hydrological year 1985 to 2008.

Stations	Mean annual Precipitation (mm)
Cervos	1,028
Chaves	576
Couto	1,422
Travancas	903

**Table 3:** Reference values used to characterize WWTP discharges in the SWAT model. Per capita values of flow and constituents concentrations according to the Portuguese legislation (Decreto-lei 236/98 and Decreto-lei 348/98).

Parameters	Units	Value
Per capita flow	L/(p.e.·day)	100
Total Suspended Solids (TSS)	mg/L	60
Ammonium	mg NH_4_/L	10
Nitrate	mg NO_3_/L	50
Total phosphorus	mg P/L	3 *

* 2 mg/L if the WWTP is located in a sensitive area and serves more than 10,000 p.e.

### 3.2. Implementation of the SWAT Model

SWAT simulation period was from 1985 to 2010 (hydrologic years). The soil parameters needed for the model implementation (e.g., hydraulic conductivity, bulk density, *etc*.) were obtained from texture information (soil map) using pedotransfer functions [33]. WWTP discharges were included in the model as point sources, with flow and nutrient loads estimated as mentioned in Section 3.1. Nutrient loads associated with the use of fertilizers were imposed assuming a maximum of 50 kgN/ha per application, in a total of 200 kgN/ha in a year. These values are in accordance with the Code of Good Agriculture Practices from the Portuguese Ministry of Agriculture.

SWAT default parameter values generate a high base-flow, thus causing groundwater to feed the riverine systems for months after the rainy season, contradicting the hydrograms that characterize the study area. As such, parameters GW_DELAY and ALPHA_BF were changed from the SWAT model default values to 3 and 1 days, respectively, so that the water retention in the soil could be decreased. Model results were validated for estimated flow, by comparing the model predictions for annual flow with field data for the 1985 to 2005 period.

### 3.3. Implementation of the CQW2 Model

Torrão reservoir was modelled for a 16 km river stretch. There are 17 computational elements of variable length (between 475–2,575 m), having a maximum number of layers at the dam wall (34). A vertical resolution of 2 m is homogenous in all domain. Bathymetric data were extrapolated from a 1:250,000 topographic chart and together with the volume accumulation curve (VAC) were used to setup the model 2D grid. VAC generated by the model was compared with the real curve from the SNIRH for accuracy. The discharge location at the dam wall was setup according to the reservoir operation information provided by SNIRH. Discharged flow was estimated based on river inflow and water volume observed at the reservoir. The nutrient load in the outflow is estimated by the model at each time instant.

The model runs for 13 years (1996 to 2009), the period with available field data for water quality parameters to assess model performance. Simulations were conducted with a 1h time step, and state variables were outputted every 48 h. The water-quality model was setup to simulate the growth of three different phytoplankton groups (diatoms, chlorophytes and cyanobacteria), with environmental conditions such as radiation, temperature and mixing processes determining the vertical distribution and growth of algal cells. Parameter values were within the range of reference values used in studies of water quality in reservoirs, and were set by the default values in the CQW2 model. Model calibration was performed by trial and error until the simulated results agreed with observed data [9].

### 3.4. Simulations Scenarios

Three distinctive scenarios were considered to assess the impact of acting on the nutrient sources: (1) the reference scenario using the results from SWAT simulations with the actual conditions, (2) a 10% nutrient load reduction in the SWAT output, and (3) an extreme scenario where WWTP were removed from the SWAT simulation. 

## 4. Results and Discussion

This study focuses on predicting the changes in the water quality of the reservoir by acting on the nutrient loads from the watershed. Firstly, the ability of the SWAT model in simulating the behavior of the system is compared to river flow data. Then the results of CQW2 are compared with field data and its performance evaluated to illustrate that the model is able to reproduce the reference scenario. Finally, it is shown how the nutrient load reduction affects the status of the system.

### 4.1. SWAT Model Validation

**Figure 4:**
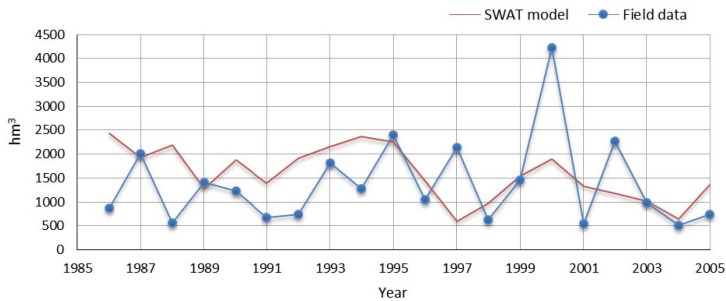
Annual flow according to SNIRH record values for Fridão hydrometric station, and estimated by SWAT model.

SWAT model estimates for river flow were validated with field data from a hydrometric station (Fridão) in the watershed from 1986 to 2005. Model results and field data are illustrated in Figure 4, and show a similar trend and approximated values. The difference of the mean total annual flow between the two series (model and data) is ~15% (1,586 hm^3^ in SWAT and 1,377 hm^3^ in field data). The differences can be attributed mostly to the fact that the hydro-electrical power generating units that regulate the flow of the Tâmega River are not considered by SWAT.

The results of SWAT simulation are compared with that of Fridão Station in terms of R^2^, root mean square error (RMSE) and Nash Sutcliffe efficiency to evaluate the goodness of fitness for the estimated and observed instant flow. This comparison was performed for the period between 2007 and 2009 because field data have increased in temporal resolution (daily values) from 2007 onward. The results are shown in Table 4 and the statistical parameters suggest a satisfactory correlation between model results and field data, denoting similar trends and an acceptable model efficiency according to the Nash-Sutcliff coefficient.

**Table 4:** Statistical parameters for the comparison of SWAT model predictions and field data from a hydrometric station located at Fridão.

Period	Mean flow (m^3^/s)		RMSE (m^3^/s)	R^2^	Nash-Sutcliffe Efficiency
	Model	Data			
2007–2009	53	48	51	0.6	0.4

### 4.2. CE-QUAL-W2 Model Performance Assessment

**Figure 5:**
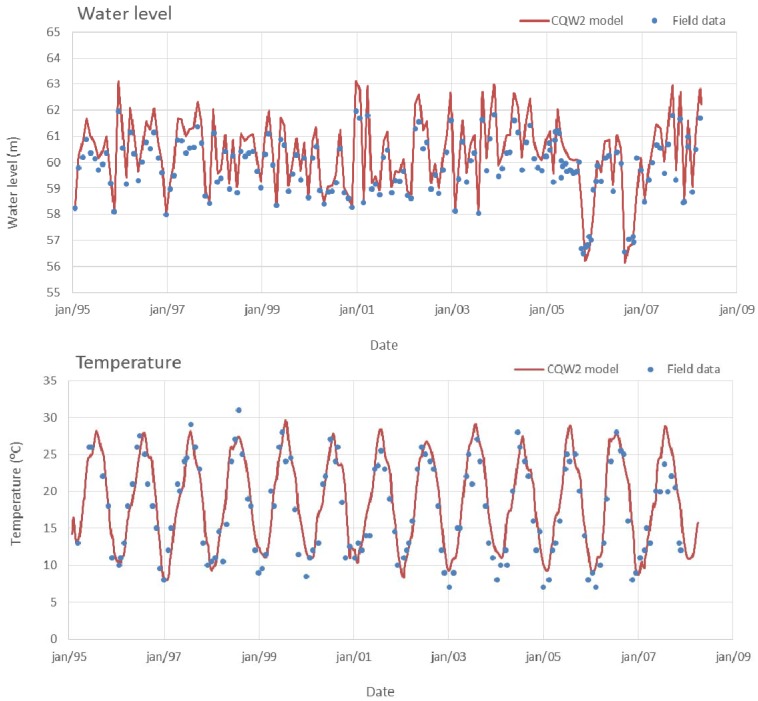
Water level and surface temperature at Torrão reservoir (model estimates and field data).

Model predictions for the reference situation at Torrão reservoir (near dam wall) and field data for water level and surface temperature are compared in Figure 5. Water levels estimated by the model deviate slightly from observed values because of the differences between real bathymetry and model domain geometry. Model results for surface temperature show that a seasonal regime is adequately reproduced, as well as the thermal amplitude, thus denoting a correct simulation of the effect of the influence of solar radiation, air temperature and wind action (vertical mixing).

**Figure 6:**
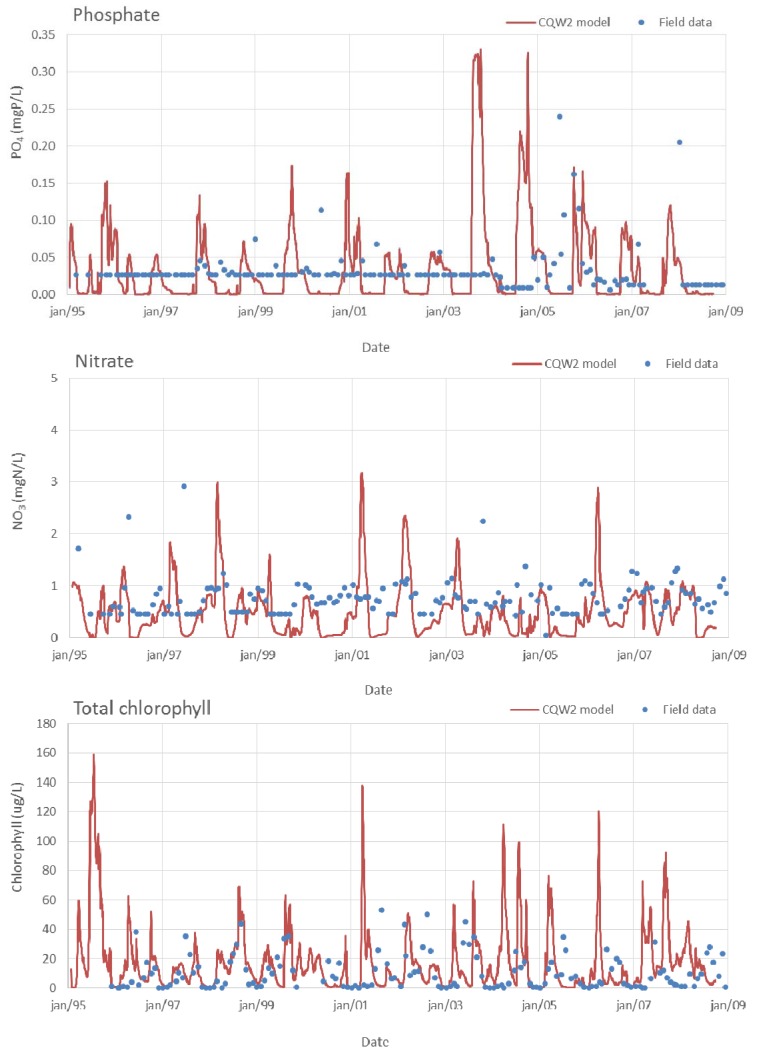
Surface nutrients and chlorophyll concentrations at Torrão reservoir (model estimates and field data).

Time series comparison between field data and model results for nutrients (PO_4_ and NO_3_) and chlorophyll are shown in Figure 6. The model reproduces the general trend of field values and their magnitude. Model results and data showed significant season and inter-annual variation in nutrient concentration, with maximum values in autumn-winter periods, when the watershed drainage increases, followed by a sharp decrease in spring months due to phytoplankton uptake.

Chlorophyll-*a in situ* data show peaks above 40 µg/L in some years (2001–2003, for example), frequently in later summer periods, and it is also possible to observe that most blooms show concentrations above the eutrophication limit of 10 µg/L (geometric mean of chlorophyll concentration at surface between April and March). Field data reveal a significant inter-annual variation, a pattern also observed in model results. Despite some over-estimated bloom peak concentrations of chlorophyll, the model captures the seasonal fluctuations and the two different blooms that occur in some years. The low temporal resolution of field data prevents any further validation of the bloom values simulated by the model.

Modelling results for Torrão reservoir in reference situation produced a geometric mean of surface chlorophyll-*a* concentration for the period 1995 to 2009 of 10.3 µg/L. The same parameter for field data was 10.6 μg/L (about 2.8% difference, see Table 5).

**Table 5:** Trophic state of the Torrão reservoir for the 1995–2009 period (based on the geometric mean of chlorophyll concentration at the surface, from April to September).

Scenario	Trophic state
Reference scenario – field data	10.6 (eutrophic)
Reference scenario – model simulation	10.3 (eutrophic)
Simulation with a 10% nutrient load reduction	9.2 (mesotrophic)
Simulation without point sources (WWTP)	4.0 (mesotrophic)

### 4.3. Pressures

Uncertainties in the modelling are inevitable because frequently *in situ* data are insufficient. In this particular case, the low temporal resolution prevents any attempt to fit the model results to field data. Nevertheless, models are effective in data poor conditions [10], and the simulation results can be considered acceptable to set water environmental management targets under the condition of limited data [9]. The validated model framework for reference situation estimated an input to Torrão reservoir of around 2,984 tonN/year being 61.5% inorganic (ammonia, nitrate, and nitrite) and 324 tonP/year with 77.8% inorganic (orthophosphate). Point sources from WWTP account for 1% of total nitrogen and 2% for phosphorus, being the remaining diffuse loads from managed areas and forestry.

### 4.4. Maximum Load to the Reservoir

The pollution load reduction required to meet the water quality objectives were obtained by simulating different scenarios of nutrient reduction. Load reduction scenarios were performed to quantify the total maximum input load that allows the reservoir to be classified as mesotrophic. Results are presented in Table 5. Model simulations showed that a load reduction of 10% to the reference situation would allow the geometric mean for surface chlorophyll-*a* at the dam wall to be reduced to 9.2 µg/L, thus changing the classification of the system from eutrophic to mesotrophic.

**Figure 7:**
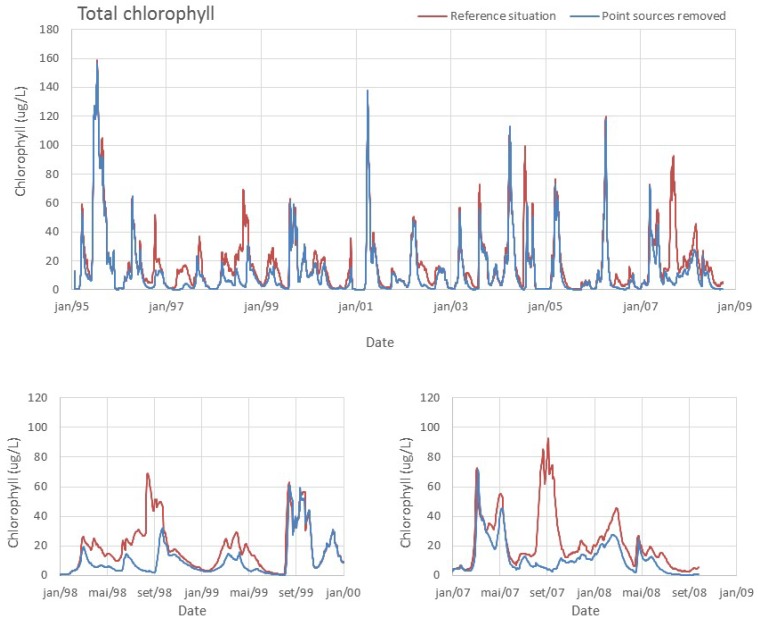
Model results for the reference situation and for the scenario where the point sources of nutrients have been removed. Lower panels show smaller periods to highlight differences in results.

**Figure 8:**
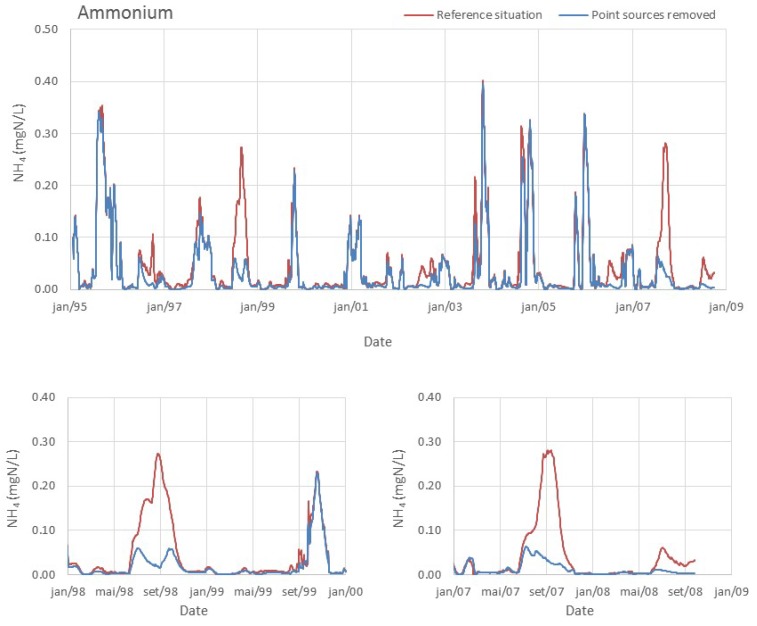
Model results for the reference situation and for the scenario where the point sources of nutrients have been removed. Lower panels show smaller periods to highlight differences in results.

The simulation for the extreme scenario of removing all point sources (WWTP), accounting for a load decrease of only 41 tonN/year and 7 tonP/year, would result in a reduction in the geometric mean of chlorophyll to approximately 4 µg/L. The significant reduction suggests that WWTP is the most significant source of nutrients that contributes for the trophic state of the reservoir. Nonetheless, land use and land cover have been identified has a major contributor of surface water pollution [34]. Interestingly, this source of nutrients only accounts for ~2% of the total nutrient input, but its contribution to phytoplankton growth seems to be critical, as seen in Figure 7. Most of the nutrients that arrive at the reservoir during the dry season are originated in WWTP (diffuse load is almost inexistent and natural river flow reduced). Despite the lower flow of their effluents, the WWTP discharges have high ammonium concentrations and constitute almost the totality of the river load in the dry season. This input seems to be stimulating and maintaining bloom conditions that occur in late spring and summer months, since the ammonium concentration is significantly lower in the scenario without the contribution of WWTP (Figure 8).

Management actions to reduce eutrophication in reservoirs are site-specific. This has already been pointed out for other reservoirs in Portugal [35]. Results for the simulated scenarios in Torrão reservoir suggest that a reduction in nutrient load originated by point sources could be enough to change the actual trophic state of the reservoir. Nevertheless, the contribution of diffuse sources to nutrient enrichment must also be considered through the implementation of better agriculture practices, since the cause-and-effect relationship between agriculture land use intensity and stream water quality has been demonstrated to be an important factor for the maintenance of stream water quality [6]. The same connections has already been proposed as the cause of eutrophication in other Portuguese reservoirs [36].

## 5. Conclusions

The primary goal of this study was to estimate pollution load reductions required to satisfy water quality objectives. A 10% reduction of nutrient inputs to Torrão reservoir seems enough to change its trophic status from eutrophic to mesotrophic. The reduction can target both point and diffuse sources of pollution by, respectively, reducing WWTP effluents and setting better agricultural practices.

Results show that the contribution of point sources is significant (mainly in dry season where point discharges dominate the input loads) and probably control the trophic status of the reservoir, despite of its relatively low percentage of annual nutrient input (around 2% in annual load but almost the total of dry season load). Their contributions seem to be crucial in summer months when river flow is low, representing the main source for primary production in the system. 

Acting on point sources may lead to a more immediate effect because soil and aquifers take longer to recover. However, a complete removal of nutrient is not feasible for all WWTP in the watershed. As such, it is suggested that actions should target effluent water quality improvements in some WWTP, but also diffuse source reduction in order to reduce the total maximum input load and mesotrophic level.

The results obtained in this study are intended to guide informed risk management decisions for managers who have water quality targets to achieve. In one way coupled watershed-reservoir models can be seen as a tool to evaluate the effectiveness of proposed operation strategies. For Portuguese reservoirs, our results reinforce previous similar observations [37,38]. Ultimately, the application of models provides a descriptive status of changed water quality for different scenarios, leaving water managers to decide adequate measures to implement to meet the suggested criteria.
